# Limb Salvage Versus Amputation: A Review of Variables Influencing the Assessment and Decision-Making in Complex Limb Fracture Management

**DOI:** 10.7759/cureus.74322

**Published:** 2024-11-23

**Authors:** Sanjeevi Bharadwaj, Subasri Balasubramanian

**Affiliations:** 1 Trauma and Orthopaedics, The Royal London Hospital, Barts Health National Health Service (NHS) Trust, London, GBR; 2 Emergency Medicine, West Midlands Deanery, Birmingham, GBR

**Keywords:** boast, complex limb injuries, ganga score, gustilo anderson classification, limb salvage versus amputation, lower extremity assessment project (leap), mangled extremity severity score, national institute for health and clinical excellence (nice), orthoplastics, scoring systems

## Abstract

Complex limb injuries are combination injuries that involve all components of a limb's tissue, such as skin, bone with its surrounding soft tissue cover, and neurovascular elements. Complex limb trauma often has a background of a significant mechanism of injuries such as high-velocity road traffic accidents, ballistic injuries, industrial accidents, and other major mechanisms of injuries which involve high amounts of energy transfer through these tissue layers. These injuries pose a major challenge to trauma and orthopaedic surgeons. This article aims to provide a comprehensive, up-to-date literature review on assessing complex limb injuries and their variables that affect decision-making and outcomes in both limb salvage and amputation. The preservation of a competent blood supply up and distal to an injury is of vital importance for any limb to survive. In a haemodynamically stable patient, it is pivotal to assess the status of peripheral circulation by examination of the distal pulses, skin colour, peripheral warmth and capillary refill time. An early and prompt multidisciplinary team approach with effective and timely input from relevant specialities while prioritising saving life to limb salvage should be a vital aspect of the initial assessment of patients with complex limb injuries. Although salvage versus amputation could be a tough decision, there are relevant scoring systems that can be used as guiding tools in the decision-making process. Although evidence suggests that amputation may result in favourable outcomes, the final decision should be based on the surgeon’s experience, the extent of soft tissue damage and the patient and/or their family's wishes after giving them an opportunity and time to process their thoughts to take the best decision in their best interest and thereby give informed and written consent. A prompt early intervention while considering the above-mentioned factors to achieve adequate soft tissue cover is the key to an optimal and functional outcome.

## Introduction and background

Complex limb injuries are combination injuries that involve all components of the tissue of a limb, such as skin, bone with its surrounding soft tissue cover and neurovascular elements. They necessitate a multidisciplinary approach for accurate, effective and timely assessment, evaluation, and management to achieve optimal functional outcomes for the patients [1]. The advancements and robust changes in emergency care of mangled extremities and ortho-plastic surgical collaboration have increased the possibility of limb salvage of injuries, which historically would have been managed by amputation [2]. These injuries are most often part of major trauma accidents and demand a holistic approach to the management of the affected patient. In a major trauma setting, the most difficult aspect of dealing with a mangled extremity injury is to decide whether to salvage or amputate the limb, which is still a matter of debate [1,2]. As long as the salvaged limb is pain-free and functional, most patients would often opt to retain it due to their functional demands, practical compulsions and cultural backgrounds [1,3].

## Review

Factors that are essential in the assessment of complex limb injuries

Complex traumatic limb injuries often have a background of a significant mechanism of injuries, such as high-velocity road traffic accidents, ballistic injuries, industrial accidents and other major mechanisms of injuries in which there is a high energy transfer. These high-risk mechanisms of injuries dictate the principle of ‘saving life before limb’, thereby necessitating an evaluation and assessment based on the principles of Advanced Trauma Life Support following the ABCDE (Airway, Breathing, Circulation, Disability, and Exposure) protocol.

It is important to reiterate that the significance of the initial assessment, evaluation and management of a patient with a complex limb injury is similar to any other patient involved in any other major trauma [4]. Detailed clinical examination is very important because these injuries are often associated with other injuries involving the head, chest, abdomen or torso as a whole [1]. The mere graphic appearance of a mangled extremity should not distract the resuscitation team from prioritising the establishment of a patent airway, optimising ventilation, and restoration of the circulation except for those which cause exsanguination, and limb injuries are seldom life-threatening in an acute setting as any ongoing bleeding from the limb wounds should be controlled by manual pressure or compression dressings till the patient is stabilised [1,4]. 

The presence of a competent blood supply distal to an injury is of extreme importance for any limb to survive. In a haemodynamically stable patient, it is pivotal to assess the status of peripheral circulation by examining the distal pulses, skin colour, peripheral warmth, and capillary refill time. Examination of sensation is crucial, as peripheral nerves are very sensitive to ischaemia, and loss of sensation is the earliest sign of a peripheral vascular compromise [1].

Reduction of a major fracture deformity or a joint dislocation followed by splinting can alleviate the distal vascular deficit. This should be followed by a documented assessment of the neurovascular status, and the presence of a weaker-than-anticipated pulse must warrant an assessment using a portable Doppler [1,4-7].

If the Doppler shows a signal reflecting the presence of a pulse, further decision to proceed with advanced vascular imaging can be taken based on the findings of an Ankle-Brachial Index (ABI) [4,8-10]. In stable patients with an ABI of <0.9 or if there is the presence of an absent or diminished pulse, it warrants the need for advanced radiological investigation such as a computed tomographic angiography, which should be performed for additional radiographic characterisation of the location and nature of the injury [4,10]. While assessing a limb with vascular injury, an on-table angiography can also aid as a useful supplement to the above investigation [2,11].

Skin and bone are usually more resistant to the impact of shrapnel or a contaminated fragment. Meanwhile, a muscle has relatively less resistance, which can result in the tracking of contamination along the fascial planes. Often, the contamination and soft tissue loss are more extensive than what is apparent initially, in which cases traditional guillotine amputations were performed in which the skin, muscle, and bone are cut at the same level. However easy and quick this may sound, the post-amputation wounds have delayed healing and wound healing problems, as the wound closure would be challenging. Moreover, the final level of the amputation is usually a bit more proximal than what is initially planned or necessary. Therefore, fashioning definitive flaps while performing the primary amputation is recommended nowadays by many surgical units, as this helps maintain stump length and, in turn, facilitates early wound closure [1,12].

An urgent operative intervention with a temporary intravascular shunt complimented with an external fixation is indicated in complex limb injuries with vascular compromise secondary to a vascular injury with absent pulses [2,13]. Depending on the clinical status of the patient, an early or delayed definitive vascular reconstruction and soft tissue cover can be planned at a later date after the blood flow has been restored [2,13].

Variables influencing the decision-making factors in salvage versus amputation for complex limb injuries

All complex limb injuries necessitate accurate and prompt assessment, evaluation, and management for achieving optimal functional outcomes for the patients as these are combination injuries that involve all components of the layers of the limb tissue, such as skin, bone with its surrounding soft tissue cover, and neurovascular elements [1].

Unfortunately, there are very few guidelines available for decision-making with regard to primary amputation versus limb salvage, and the existing information revolves around controversial, confusing, and conflicting with a lack of level I studies [1]. 

Salvage versus amputation for complex limb injuries involves a major decision-making process that incorporates the limb salvageability and the patient’s high expectations while considering possibilities of limb salvage in a certain situation and whether the patient would benefit from the salvaged limb to achieve a functional restoration, [1] as such major procedures involve repeated revision procedures and longer hospital stay [1,14-16].

Although it is a natural tendency for a surgeon and the patient to salvage a badly injured limb or a mangled extremity at any cost, there are many factors which have to be taken into account in the decision-making process [1,3,17]. These factors play an important role in making the crucial decision of salvage versus amputation for complex limb injuries or mangled extremities [3,17].

Scoring systems and their role in decision-making

Although the most commonly used classification is the Gustilo-Anderson Classification system [18], this classification does not give any guidance on the management of complex mangled extremity injuries apart from the use of antibiotics and the involvement of relevant specialities while treating such complex injuries. It also does not offer any guidance concerning the comorbid components nor assess the need for salvage, as it was designed to guide us about the need for soft tissue cover [17-19].

There have been many scoring systems that have been published over the last couple of decades, such as the Mangled Extremity Severity Score (MESS) [1,20,21], the Limb Salvage Index (LSI) [1,22], the Predictive Salvage Index (PSI) [1,23], and the Nerve Injury, Ischemia, Soft Tissue Injury, Skeletal Injury, Shock and Age (NISSSA) of the patient score [1,24], and the Hannover Fracture Scale-97 (HFS-97) [1,25]. All these scoring systems had been specifically designed to evaluate and assess complex combined orthopaedic and vascular injuries [1]. A comparison of the different scoring systems is shown in Table 1.

**Table 1:** Comparison of the components of the different scoring systems GHOISS: Ganga Hospital Open Injury Severity Score; LSI: Limb Salvage Index; MESI: Mangled Extremity Syndrome Index; MESS: Mangled Extremity Severity Score; NISSSA: Nerve Injury, Ischemia, Soft Tissue Injury, Skeletal Injury, Shock and Age; PSI: Predictive Salvage Index Source: Akgun Demir and Karsidag, 2020 [26]

	MEIS	MESS	PSI	LSI	NISSSA	GHOISS
Age	+	+			+	+
Shock	+	+			+	+
Warm Ischemic Time	+	+	+	+	+	+
Bone Injury	+		+	+		+
Muscle Injury			+	+		+
Skin Injury	+			+		+
Nerve Injury	+			+	+	
Deep Vein Injury				+		
Skeletal/Soft Tissue		+			+	
Contamination					+	+
Time to Treatment	+			+		
Comorbidity	+					+

The MESS (see Table 2) was devised to aid as an outcome predictor, especially for the complex mangled limb injuries sustained after polytrauma [1,21]. This was based on four variables: skeletal/soft tissue injury, limb ischaemia, shock and patient age [17,20,21,27]. Many studies by various groups have concluded and recommended that a complex limb injury with irreversible ischemia or gangrene with a MESS >7 was an absolute indication for amputation [17,20,27].

**Table 2:** Mangled Extremity Severity Score *Score doubled for ischemia >6 hours. GSW: gunshot wounds; MVA: motor vehicle accidents The Mangled Extremity Severity Score (MESS) designed by Johansen et al. [20]

Mangled Extremity Severity Score (MESS) criteria
A. Skeletal/soft-tissue injury
1. Low energy (stab; simple fracture; pistol gunshot wound)
2. Medium energy (open or multiple fractures, dislocation)
3. High energy (high-speed MVA or rifle GSW)
4. Very high energy (high-speed trauma + gross contamination)
B. Limb ischemia
1. Pulse reduced or absent but perfusion normal*
2. Pulseless; paresthesia, diminished capillary refill
3. Cool, paralyzed, insensate, numb*
C. Shock
0. Systolic BP always > 90 mmHg
1. Hypotensive transiently
2. Persistent hypotension
D. Age (years)
0. <30
1. 30-50
2. >50

Although factors like delay in revascularisation beyond six to eight hours, popliteal artery injuries and ligated arteries in the presence of associated multiple fractures have been considered poor predictors of limb salvage, [1] some recent studies have shown that there is a possibility of achieving a successful limb salvage along with a good functional outcome in patients with a MESS >7 [1,28-30].

However, the studies that followed showed that the above-mentioned scores could not be considered effective outcome predictors in type IIIB open injuries where vascularity was intact, as these scores had poor sensitivity and specificity [1,31]. Since there is a high incidence of fracture non-union, infections resulting in a prolonged period of treatment and repeated procedures lead to poor functional outcomes and secondary amputations, which makes it very challenging to manage type IIIB open injuries of the limb, thereby necessitating the need for a scoring system which can guide us by providing treatment guidelines while being an outcome predictor of prognosis and survival of the affected limb. This has also been concluded by major studies that evaluated the Gustilo-Anderson classification system (see Table 3) [1,14,32-39]. In type IIIB open injuries where vascularity was not compromised, the severity of muscle damage and the amount of bone loss can guide in salvage versus amputation [1]. As a predictor of final functional outcome and limb salvage, the MESS scoring system was not as effective in predicting primary amputation [40].

**Table 3:** The modified Gustilo-Anderson classification of open fractures

Type	Description
I	A fracture with a clean laceration <1 cm in length, caused by low-velocity trauma and minimal contamination
II	A fracture with a laceration >1 cm in length lacking any severe soft tissue damage or devitalized tissue
III	A fracture with extensive soft-tissue loss
IIIA	Adequate coverage of fracture by soft tissue despite extensive cutaneous laceration or flaps, or high-energy trauma regardless of the size of the wound
IIIB	More extensive injury and contamination of the soft tissue, periosteal stripping and soft-tissue gaps present with exposed bone; will likely require either a local soft-tissue flap or a free flap for coverage
IIIC	Any open fracture with an arterial injury requires repair regardless of the degree of soft-tissue disruption

To address the limitations of MESS and to overcome the disadvantages of Gustilo’s classification, the Ganga Hospital Open Injury Severity Score (GHOISS) was developed in 1994. This scoring system could accurately predict salvage in open injuries where there was no vascular compromise in type IIIB open injuries while having high sensitivity, specificity, positive and negative predictive values, as well as a high interobserver agreement which was independent of experience [1,19,19].

However, the Lower Extremity Assessment Project (LEAP), which was a multicentre study of severe lower extremity trauma, was conducted by Professor Ellen MacKenzie from Johns Hopkins University across eight level 1 trauma centres in the USA. The project was conducted in an attempt to authenticate these scoring systems, following which they concluded that the clinical utility of the five scoring systems mentioned above (MESS, PSI, LSI, NISSSA, HFS-97) could not be verified in predicting amputation as these scoring systems were inaccurate, unreliable and had low sensitivities [1,5,17,19,27]. It also concluded that there was no improvement in the outcome of the patient group at two and seven years over a seven-year follow-up and that the long-held belief that the absence of sensation in the plantar aspect of the foot was an indication for amputation was no less than a myth [1,5,6,17,19,27]. However, it is important to note that this study did not set any criteria for salvage versus amputation, which leaves the decision-making for limb salvage versus amputation to the clinicians to make a judicious decision taking the combination of factors that have been discussed above. The sensitivity and specificity of the five scoring systems of the LEAP study are shown in Table 4 [30].

**Table 4:** Sensitivity and specificity of the predictive scoring systems adapted from the LEAP study HFS-97: Hannover Fracture Scale-97; LEAP: Lower Extremity Assessment Project; LSI: Limb Salvage Index; MESS: Mangled Extremity Severity Score; NISSSA: Nerve Injury, Ischemia, Soft Tissue Injury, Skeletal Injury, Shock and Age; PSI: Predictive Salvage Index

	MESS	PSI	LSI	NISSSA	HFS-97
Sensitivity	0.45	0.47	0.51	0.33	0.37
Specificity	0.93	0.84	0.97	0.98	0.98

Moreover, a systematic review by Schiro et al. showed that the utility of the scoring systems in predicting functional outcomes or determining salvage versus amputation is very limited and cannot be of much practical use [2,7].

Current guidelines by the National Institute for Health and Clinical Excellence (NICE) recommend that limb salvage versus amputation should not be decided based on an injury severity tool [2]. However, for a life-threatening uncontrolled haemorrhage, NICE recommends an emergency amputation [2]. At the same time, a delayed primary amputation should be performed within 72 hours of the injury in cases where there is no threat to the patient's life [2,6].

Above all, it is important to reiterate the importance of multidisciplinary assessment and decision-making with the involvement of the ortho-plastics team, rehabilitation team, patients, and their relatives, as recommended by both NICE and British Orthopaedic Association Standard for Trauma (BOAST) guidelines [2].

## Conclusions

Complex limb injuries are combination injuries that involve all components of a limb. Detailed clinical examination is very important. The existing guidelines and various open injury severity scoring systems are confusing and controversial, although a few of them have still been actively in use. However, the studies that followed showed that these scoring systems could not be considered effective outcome predictors for all complex open limb injuries, as these scores had poor sensitivity and specificity. Therefore, the decision of salvage versus amputation in a complex extremity trauma should be taken after careful consideration of all the above-discussed factors. A prompt early intervention while considering the above-mentioned factors to achieve adequate soft tissue cover is the key to an optimal and functional outcome.
